# Cannonball Lesions: A Case Report

**DOI:** 10.7759/cureus.82554

**Published:** 2025-04-19

**Authors:** Robin Sia

**Affiliations:** 1 General Medicine, Austin Hospital, Heidelberg, AUS

**Keywords:** cancer, cannonball, hepatitis, hepatocellular, malignancy, medicine, metastases, palliative, pulmonary

## Abstract

Cannonball lesions refer to multiple, well-defined, round pulmonary metastases seen on chest radiographs or CT scans. They are most commonly associated with hematogenous spread of malignancies, most frequently linked to renal cell carcinoma, choriocarcinoma, colorectal cancer, and breast cancer. We report a case of cannonball pulmonary metastases secondary to hepatocellular carcinoma (HCC).

## Introduction

Hepatocellular carcinoma (HCC) is a primary liver malignancy commonly seen in patients with chronic hepatitis B virus (HBV) infection and cirrhosis. HCC is the most prevalent type of liver cancer, accounting for roughly 90% of cases globally, with hepatitis B infection (HBV) being the most common risk factor, accounting for 50% of cases [1]. It poses a significant public health challenge and is currently recognized as the fastest-rising cause of cancer-related deaths in the United States. If this trajectory continues, HCC is projected to become the third leading cause of cancer-related mortality by 2030 [1]. While HCC primarily metastasizes to the lungs, bone, and adrenal glands in 13.5-42% of cases, extensive pulmonary involvement presenting as cannonball metastases is rare and often signifies advanced disease [2]. Furthermore, it is estimated that 50% of HCC cases are diagnosed incidentally, demonstrating the need for continued screening [1].

We discuss a rare case of a 62-year-old female with chronic HBV and cirrhosis with newly diagnosed HCC presenting as pulmonary cannonball metastases. This case further highlights the critical role of routine HCC surveillance in at-risk patients to facilitate early detection and improve outcomes.

## Case presentation

**Figure 1:**
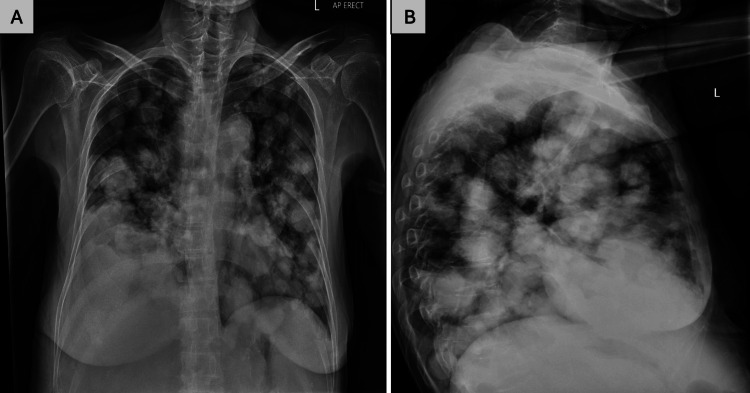
Chest radiography images showing multiple large and round nodules

A 62-year-old female with chronic HBV and liver cirrhosis, previously on tenofovir, presented to the emergency department with progressively worsening shortness of breath over three months. A chest X-ray revealed numerous well-circumscribed, bilateral pulmonary opacities, consistent with “cannonball” metastases (Figures 1A, 1B). She had been previously monitored in the liver clinic with liver ultrasound, serological markers including HBV viral load and serum alpha-fetoprotein (AFP) levels, which had been normal; however, she subsequently had issues with compliance and had been lost to follow-up for approximately two years, missing HCC surveillance. A month prior, she had sought care at another hospital for right flank pain. Serology with AFP levels of 293 ng/mL (<16 ng/mL) and imaging, including an abdominal CT scan and liver biopsy, confirmed HCC. The presence of cannonball lesions suggested hematogenous spread of the malignancy to the lungs, likely from HCC. Given the advanced metastatic disease and the patient's and family's preferences, she was transitioned to palliative care. This case underscores the importance of consistent HCC surveillance in patients with chronic HBV and cirrhosis to facilitate early detection and timely management of malignancy.

## Discussion

Cannonball lesions refer to multiple, well-defined, round pulmonary metastases seen on chest radiographs or CT scans. They are most commonly associated with hematogenous spread of malignancies, usually seen in the setting of renal cell carcinoma and choriocarcinoma. In rare cases, pulmonary metastases with the same appearance may be secondary to synovial sarcoma, endometrial carcinoma, or HCC [3]. Our case highlights the rarity of pulmonary cannonball metastases in the setting of HCC.

Cannonball lesions are indicative of widespread metastatic disease and often have a poor prognosis depending on the primary tumor [2]. In terms of infectious causes, cannonball lesions can represent fungal infections such as histoplasmosis, coccidioidomycosis, tuberculosis, and hydatid disease secondary to echinococcus [4,5]. Cannonball lesions can also be due to autoimmune diseases such as granulomatosis with polyangiitis, rheumatoid arthritis in the form of rheumatoid nodules, and sarcoidosis, although this usually occurs in a more interstitial pattern [4]. In terms of its radiographic appearance, it is most often seen on chest X-rays or CT scans. Cannonball lesions can vary widely, from a few millimeters to several centimeters, and are usually bilateral, scattered throughout the lung fields with smooth and well-defined margins [6].

The clinical evaluation of cannonball lesions begins with a comprehensive and focused history, as it provides essential context for interpreting imaging findings. A detailed assessment should include any known history of malignancy, especially tumors prone to hematogenous pulmonary spread, such as renal cell carcinoma, testicular cancer, choriocarcinoma, or sarcomas. Additionally, clinicians should inquire about travel history, particularly to regions endemic for fungal infections like histoplasmosis or coccidioidomycosis, and assess for risk factors related to tuberculosis or parasitic infections. Signs or symptoms of autoimmune disease, such as rheumatoid arthritis or vasculitis, should also be explored as they may rarely present with similar pulmonary findings [4].

Imaging plays a central role in evaluating cannonball lesions. A contrast-enhanced CT scan of the chest is typically the next step after initial detection on chest X-ray, as it provides detailed anatomical and morphological information about the lesions. In cases where malignancy is suspected, a PET-CT scan may be used to assess metabolic activity of the lesions, identify the primary tumor if unknown, and evaluate for further metastases [6]. Imaging findings can help narrow the differential, but are rarely diagnostic on their own. To establish a definitive diagnosis, histological confirmation with immunohistochemistry via biopsy is often required. For peripheral nodules, a CT-guided percutaneous lung biopsy is preferred, while bronchoscopic biopsy may be suitable for central lesions or when multiple sampling options are needed. In some cases, particularly when previous biopsies are non-diagnostic or lesions are not easily accessible, endobronchial ultrasound (EBUS) or surgical options such as video-assisted thoracoscopic surgery (VATS) may be considered [7]. Histopathology, along with microbiologic cultures and molecular tests, ultimately determines whether the cause is neoplastic, infectious, or inflammatory.

Immunotherapy-based combination therapies have emerged as the preferred first-line treatment for advanced HCC, due to their enhanced efficacy and promising survival benefits. The role of cytotoxic chemotherapy, historically associated with limited effectiveness in HCC, has declined with the development of novel immunotherapeutic and molecularly targeted treatments. Nevertheless, chemotherapy remains a consideration for patients who are not candidates for other therapeutic options [8]. In 2022, the Barcelona Clinic Liver Cancer (BCLC) guidelines identified atezolizumab combined with bevacizumab (Atezo-Bev) as the preferred first-line treatment for advanced HCC in patients with Child-Pugh A liver function and no high-risk features for variceal bleeding [9]. Metastasectomy may be considered in select cases of pulmonary metastases; however, the role in HCC is not well-established. Eligibility depends on the patient’s surgical risk profile, along with specific tumor characteristics such as number, size (typically <3 cm), and location of lesions. However, due to the frequently multifocal nature of pulmonary metastases in HCC, surgical resection is often not feasible [8].

The overall prognosis for HCC remains poor, with a five-year survival rate of approximately 18%. In cases of metastatic disease, this rate drops significantly, with only around 2% of patients surviving beyond five years [8,10]. Therefore, vigorous surveillance for HCC in patients with chronic HBV infection using ultrasound with or without AFP every six months is strongly recommended [11].

## Conclusions

This report highlights the critical need for regular HCC surveillance in patients with chronic hepatitis B and cirrhosis. Despite previous monitoring, our patient's loss to follow-up resulted in delayed diagnosis of HCC, which had already metastasized to the lungs by the time it was discovered. The presence of cannonball metastases indicated hematogenous spread of the malignancy, underlining the aggressive nature of HCC when not detected and managed early. Given the advanced metastatic disease, the patient was transitioned to palliative care, in alignment with her and her family’s wishes. This case highlights the importance of ongoing monitoring in high-risk populations to prevent such delays in diagnosis and optimize treatment outcomes.
